# Advances in succinic acid production: the enhancement of CO_2_ fixation for the carbon sequestration benefits

**DOI:** 10.3389/fbioe.2024.1392414

**Published:** 2024-03-28

**Authors:** Fanzhen Lin, Wenwei Li, Dan Wang, Ge Hu, Zhao Qin, Xue Xia, Lin Hu, Xuemei Liu, Ruoshi Luo

**Affiliations:** Department of Chemical Engineering, School of Chemistry and Chemical Engineering, Chongqing University, Chongqing, China

**Keywords:** succinic acid, CO_2_ sequestration, waste biomass, Bioelectrochemical, Bioreactors, high-throughput screening

## Abstract

Succinic acid (SA), one of the 12 top platform chemicals produced from biomass, is a precursor of various high value-added derivatives. Specially, 1 mol CO_2_ is assimilated in 1 mol SA biosynthetic route under anaerobic conditions, which helps to achieve carbon reduction goals. In this review, methods for enhanced CO_2_ fixation in SA production and utilization of waste biomass for SA production are reviewed. Bioelectrochemical and bioreactor coupling systems constructed with off-gas reutilization to capture CO_2_ more efficiently were highlighted. In addition, the techno-economic analysis and carbon sequestration benefits for the synthesis of bio-based SA from CO_2_ and waste biomass are analyzed. Finally, a droplet microfluidics-based high-throughput screening technique applied to the future bioproduction of SA is proposed as a promising approach.

## 1 Introduction

Succinic acid (SA) is normally an intermediate metabolite of the tricarboxylic acid (TCA) cycle. As a C4 platform chemical, SA is the precursor for the synthesis of a variety of high value-added chemicals or materials, such as 1,4-butanediol (1,4-BDO), γ-butyrolactone, tetrahydrofuran, adipic acid, *N*-methyl pyrrolidone, and linear aliphatic esters (Kang et al., 2014; Tapin et al., 2020; Le and Nishimura, 2021). The most notable application of SA is that it could be used as a monomer for the production of biodegradable polybutylene succinate (PBS), which has a market value of $110 million (Amulya and Venkata Mohan, 2022). Therefore, the market demand for SA is huge. Traditionally, SA is chemically synthesized by maleic anhydride from petrochemical feedstocks, and Ni- or Pd-based catalysts have always been used to hydrogenate maleic anhydride into SA (Geyer et al., 2017; Zhuang et al., 2023). Despite the high conversion rate, there are still many drawbacks, which include complex operations, expensive catalysts, and serious pollution (Louasté and Eloutassi, 2020; Yao Zhang, 2020). Additionally, petroleum resources are non-renewable, and the burning of fossil fuels emits huge amounts of greenhouse gases, especially CO_2_, which causes a detrimental global warming (Martins et al., 2021). At the end of 2020, approximately 34.8 billion tons of CO_2_ were emitted from fossil fuels (Karakurt and Aydin, 2023). As a consequence, many scientists worldwide are endeavoring to explore green biosynthetic methods to obtain SA from waste and renewable feedstocks. Among these strategies, microbial fermentation for SA with utilization of CO_2_ and waste biomass as feedstocks is considered the most advantageous technology due to its simple biological operating conditions, mild enzyme-catalyzed process, and efficient CO_2_ fixation capacity. Meanwhile, CO_2_ biosequestration by microorganisms contributes to net zero emissions and is beneficial to obtaining carbon sequestration benefits (Ma et al., 2022; Song et al., 2024).

The biosynthesis of SA opens a novel pathway to utilize the greenhouse gas. Under anaerobic conditions, many wild-type strains, such as *Actinobacillus succinogenes* (Putri et al., 2020), *Saccharomyces cerevisiae* (Yan et al., 2014), *Yarrowia lipolytica* (Li et al., 2022), and *Escherichia coli* (Olajuyin et al., 2019), could utilize CO_2_ under the action of enzymes and accumulate SA (Priyadharsini et al., 2022). Theoretically, for 1 g of SA produced, 0.373 g of CO_2_ could be fixed (Amulya and Venkata Mohan, 2022). Thus, bio-SA is actually a kind of iconic CO_2_ fixation product through biomanufacturing, which helps human society to reach its global CO_2_ emissions reduction target (Belbute and Pereira, 2020). Furthermore, SA productivity could be enhanced by metabolic engineering or synthetic biology strategies, which also lead to increased CO_2_ fixation rates (CFRs) and higher carbon sequestration benefits. Moreover, the raw feedstocks for biosynthesis are less costly and more easily available than those for chemical synthesis. Renewable biomass resources, especially waste biomass such as sugarcane bagasse (Xu et al., 2021), corncob (Zhao et al., 2016), cassava root, *etc* (Thuy et al., 2017), are potential feedstocks for the production of SA and the reduction of carbon emissions. When compared to fossil-fuel-based SA, each ton of bio-based SA produced by biomass is predicted to reduce CO_2_ emissions by 4.50–5.00 tons (Gunnarsson et al., 2014).

**FIGURE 1:**
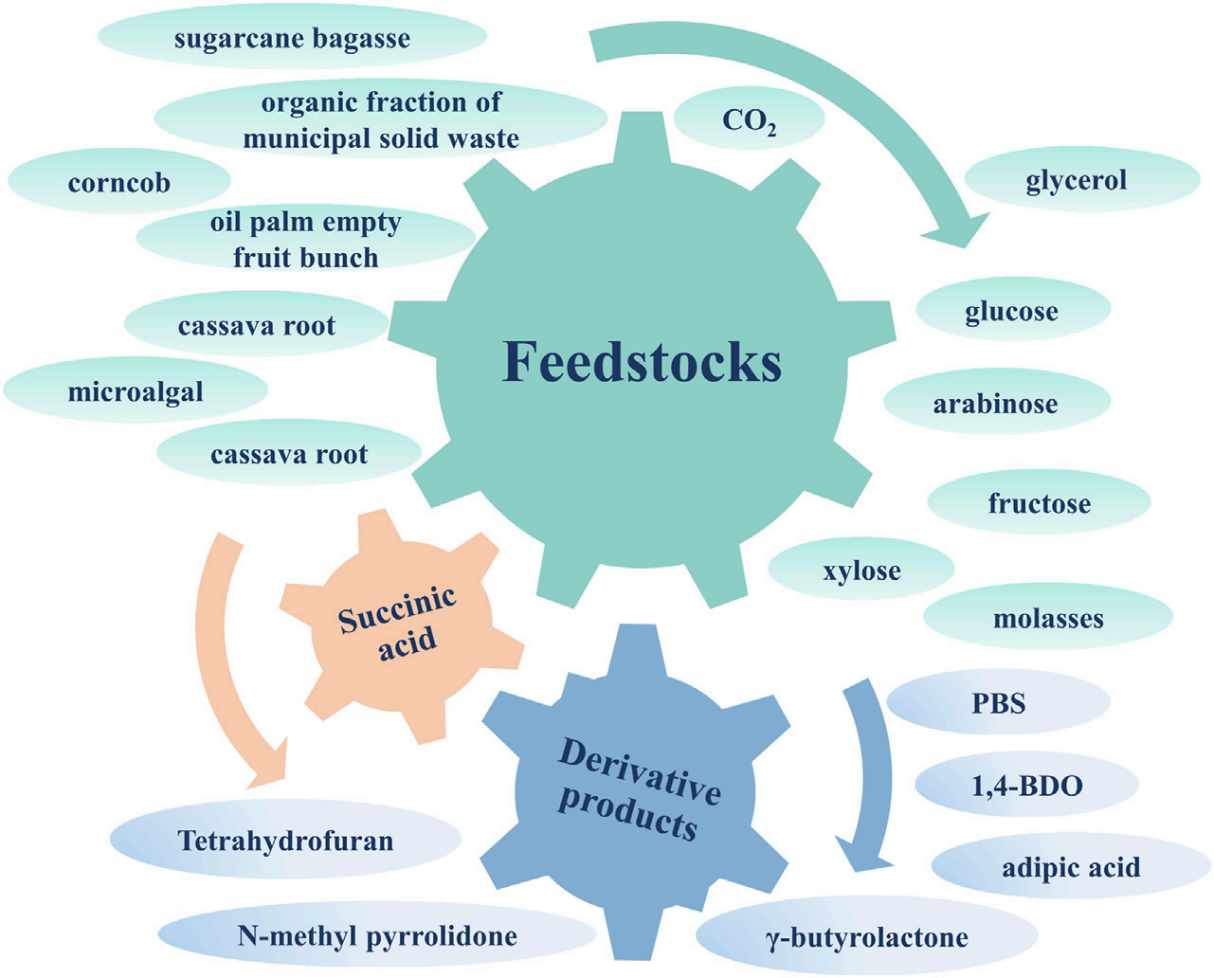
The extensive sources and product applications of SA.

In summary, the biosynthesis of SA can use CO_2_ and waste biomass hydrolysate to achieve higher carbon sequestration benefits and lower production costs, showing excellent environmental and economic benefits. This article reviews the current advances in CO_2_ fixation and waste biomass utilization during SA biosynthesis for carbon sequestration benefits. The extensive sources for SA fermentation and derivative products are shown in Figure 1. And the importance and possible improvements of efficient applied potential systems constructed by bioelectrochemical strategy and off-gas utilization through coupling bioreactors are summarized. In addition, the techno-economic analysis and the evaluation of carbon sequestration benefits for SA biorefineries are presented, which is conducive to advancing the industrialization of SA biosynthesis. Finally, a droplet microfluidics-based high-throughput screening technique applied to the future bioproduction of SA is proposed as a promising approach.

## 2 Enhanced CO_2_ fixation in SA biosynthesis

**FIGURE 2:**
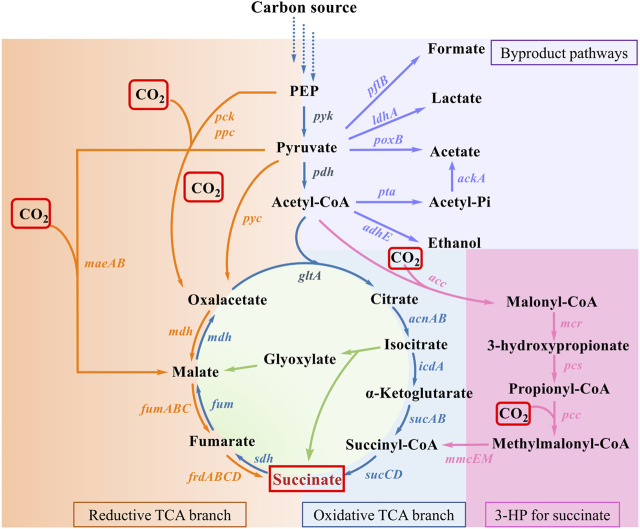
SA production pathways in microorganism. Genes of enzymes related to the pathways are presented next to the arrows, and each pathway is highlighted in a distinct color. Virtually, CO_2_ fixation occurs in reductive TCA branch and 3-HP for succinate. Abbreviations and genes: PEP, phosphoenolpyruvate; *ackA*, acetate kinase; *acnAB*, aconitase; *adhE*, alcohol dehydrogenase; *gltA*, citrate synthetase; *icdA*, isocitrate dehydrogenase; *ldhA*, lactic dehydrogenase; *maeAB*, malic enzyme; *mdh*, malate dehydrogenase; *pck*, PEP carboxykinase; *pflB*, pyruvate formatelyase; *poxB*, pyruvate oxidase; *ppc*, PEP carboxylase; *pta*, phosphotransacetylase; *pyc*, pyruvate carboxylase; *sdh*, succinate dehydrogenase; *sucABCD*, succinyl-CoA synthetase; *acc*, acetyl-CoA carboxylase; *mcr*, malonyl-CoA reductase; *pcs*, propionyl-CoA synthase; *pcc*, propionyl-CoA carboxylase; *mmcEM*, methylmalonyl-CoA epimerase and mutase; *fumABC*, fumarate hydratase; *frdABCD*, succinate dehydrogenase.

To date, a variety of natural or artificial SA biosynthesis pathways have been reported, of which four major metabolic pathways are often adopted, as shown in Figure 2 (Liu et al., 2022): 1) reductive TCA cycle; 2) oxidative TCA cycle; 3) the glyoxylate pathway; 4) the 3-hydroxypropionate cycle (3HP cycle) (Sanchez et al., 2005; Ahn et al., 2016; Vuoristo et al., 2016). The reductive TCA cycle is used by prokaryotes, such as *E. coli*, to produce SA and utilize CO_2_ (Choi et al., 2022). The beneficial effects of CO_2_ fixation and SA biosynthesis are reciprocal. On the one hand, CO_2_ can be fixed by multiple enzymes during the pathway of SA production, such as phosphoenolpyruvate carboxylase (PEPC), phosphoenolpyruvate carboxykinase (PCK), pyruvate carboxylase (PYC), malic acid enzyme (MAE), and so on. On the other hand, CO_2_ supply conditions also influence SA productivity and yield. In this section, the functions of enzymes involved in CO_2_ fixation during the production of SA are summarized, and the metabolic engineering strategies for the construction of engineered SA-producing strains are discussed. The increased benefits of microbial carbon sequestration are reflected in increased CFRs. As shown in Table 1, the efficiency of different pathways for immobilizing CO_2_ in the production of SA was listed.

**TABLE 1:** Comparison of CO_2_ fixation by different pathways of SA biosynthesis.

Pathways	CO_2_-fixing enzymes	Reaction steps	CO_2_/SA (mol/mol)	SA yield (g/g)	SA titer (g/L)	SA productivity (g/L/h)	References
The reductive branch of the TCA cycle (RTCA)							
RTCA-PPC	PPC	4	−1	1.24	89.4	1.24	Yu et al. (2016)
RTCA-PCK	PCK	4	−1	0.819	41.1	0.820	Zhang et al. (2009)
RTCA-PYC	PYC	5	−1	1.10	152	0.950	Chung et al. (2017)
RTCA-MAE	MAE	4	−1	0.202	1.82	0.0758	Kwon et al. (2007)
The oxidative branch of the TCA cycle (OTCA)							
OTCA	—	5	+2	0.610	58.3	0.990	Lin et al. (2005)
The glyoxylate pathway (GAC)							
GAC	—	3	0	0.892	95.9	0.908	Zhu et al. (2014)
The 3-hydroxypropionate cycle (3HP)							
3HP-ACC-PCC	ACC, PCC	6	−2	0.0800	2.66	0.0600	Liu et al. (2020)

Notes: RTCA, reductive TCA, cycle; OTCA, oxidative TCA, cycle; GAC, the glyoxylate pathway; 3HP, the 3-hydroxypropionate cycle; PPC, phosphoenolpyruvate carboxylase; PCK, phosphoenolpyruvate carboxykinase; PYC, pyruvate carboxylase; MAE, malic enzyme; ACC, acetyl-CoA carboxylase; PCC, propionyl-CoA carboxylase. The values of yield, titer, and productivity in the table were concluded with glucose as a substrate.

It could be concluded that the main factors influencing the CO_2_ fixation rates and SA yield are as follows: the direction of central carbon metabolic flow, the activity of key enzymes in the reductive branch of TCA cycle, the solubility of CO_2_, and the assistance of transporters and cofactors.

The knockout of genes associated with by-product production is an effective strategy to reduce byproduct accumulation and increase the flow of central carbon metabolism to the reductive TCA cycle, which utilizes CO_2_. For instance, knockout of genes encoding lactate dehydrogenase (*ldhA*), pyruvate formate lyase (*pflB*), phosphotransacetylase (*pta*), and acetate kinase (*ackA*), all of which are effective in increasing SA production and CO_2_ fixation rates (Chatterjee et al., 2001; Jiang et al., 2010). This type of engineered strain, with relevant gene deletions in the by-product pathways, laid the foundation for the subsequent increase in SA production and CFRs.

Along with the endeavor to eliminate by-products, the identification and enhancement of the key enzymes involved in the reductive branch of the TCA cycle, such as malate dehydrogenase (MDH) and PYC, were also investigated (Olajuyin et al., 2019; Ahn et al., 2020). The conversion of OAA to malate, catalyzed by MDH under anaerobic conditions, is a crucial step for SA biosynthesis (Valle et al., 2021). The catalytic characteristics of MDH from different sources were investigated, and the optimal one, *Corynebacterium glutamicum* MDH (*Cg*MDH), was introduced into *Mannheimia succiniciproducens* to develop a high-performance strain for SA production. The strain expressing *cgmdh* produces 134 g/L of SA, with a maximum CFR of 7.95 g/L/h, in a high-inoculum fed-batch fermentation, proving the importance of pathway reconstruction in strain development (Ahn et al., 2020). By selecting the gene of the key enzyme with higher enzyme activity and introducing it into the engineered strain, the yield of SA could be further improved.

The improvement of comprehensive metabolic performance usually requires the regulation of multiple metabolic pathways, and the most commonly used method is multi-enzyme co-expression. Overexpression of carbonic anhydrase (CA) and PEPC in *E. coli* DC1515 (*ΔpflA*, *ΔldhA*, and *ΔptsG400*) increased SA production from 0.750 to 16.3 g/L, and CFR increased 21.8-fold, which was 0.127 g/L/h (Wang et al., 2015; Huang et al., 2019). Additionally, overexpression of CA and PCK in *Corynebacterium acetoacidophilum* (*ΔldhA*) increased SA production from 24.0 to 27.9 g/L and CFR from 0.299 to 0.347 g/L/h (Qian and Zheng, 2023). Notably, the direct substrate of carboxylases is not CO_2_ but HCO_3_^− (^ Craveiro et al., 2022; Zaidi et al., 2022 ^)^. CA could accelerate the conversion of CO_2_ to bicarbonate, so its expression could increase the solubilization of CO_2_ in intracellular fluids so that enzymes such as PEPC or PCK could make better use of HCO_3_^−^. Although multi-enzyme co-expression is a successful strategy for SA production and CO_2_ utilization, the compatibility of each enzyme needs to be considered in practical applications (Wang et al., 2015; Hou et al., 2019).

Furthermore, the production of SA was also affected by global regulatory factors, NADH/NAD^+^ ratio, and ATP level. Although the inactivation of *pfl* and *ldh* enhances the conversion of PEP to OAA, it also restricts the regeneration of NAD^+^ from NADH produced during glycolysis and causes an excessive buildup of pyruvate. Nicotinic acid phosphoribosyltransferase (NAPRTase) is a rate-limiting enzyme of the NAD(H) synthesis system, and the overexpression of NAPRTase is conducive to the generation of NAD^+^ (Marletta et al., 2015). Therefore, the co-expression of NAPRTase and PYC in *pflB*, *ldhA*, and *ppc* deletion strains can reduce NADH/NAD^+^ ratio and redistribute the carbon flux. It resulted in a production of 12.1 g/L of SA and a CFR of 0.0835 g/L/h under anaerobic conditions (Liu et al., 2013). NAD^+^ could serve as an electron acceptor during anaerobic fermentation, while excessive energy will burden metabolism (Zhang et al., 2016). Li et al. overexpressed the soluble fumarate reductase from *S. cerevisiae* to regulate the NADH/NAD^+^ ratio and ATP level, and the SA yield reached 31.9 g/L, an increase of 39.0% compared to the control (Li J. et al., 2017). It has been proven that Cra (catabolite repressor/activator) vitally participates in the global regulation of carbon metabolism-related genes (Ishihama, 2010). Cra activates genes involved in PEP carboxylation and suppresses genes associated with glycolytic pathways (Cozzone and El-Mansi, 2005). Zhu et al. firstly constructed random mutagenesis libraries of *cra* by error-prone PCR and then determined the relative activity of key enzymes involved in SA metabolism. After integrating mutation sites, the optimal mutant strain finally produced SA 79.8 g/L (Zhu et al., 2016). Recently, reconfiguration of the reductive SA biosynthesis pathway in mitochondria by coupling oxidative and reductive TCA cycles for NADH regeneration was reported in the strictly aerobic yeast *Yarrowia lipolytica*, which produced 112 g/L SA in 62 h by batch replenishment fermentation at low pH in a yield of 0.790 g/g glucose (Cui et al., 2023).

During the process of SA synthesis, transporters responsible for C4-dicarboxylates transport could increase the metabolic flux and SA yield. Li et al. employed three SA transporters from different organisms in *E. coli* AFP111 to construct three engineered strains, and the maximum SA production could reach 68.7 g/L (Li X. et al., 2017). The results demonstrated that the efflux capacity of the engineered strain was still strong under a high concentration of SA. By overexpressing *mgtA*, which encodes the magnesium transporter, Wang et al. constructed an engineered *E. coli* strain to produce SA with MgCO_3_ or Mg(OH)_2_ as an alkaline neutralizer in fermentation. The final fermentation yield was increased to 0.860 g/g total sugar of SA with a CFR of 0.802 g/L/h (Wang J. et al., 2014). Mg^2+^ is an essential ion for numerous physiological processes, but the molecular mechanisms of Mg^2+^ channel and transporter regulation that maintain Mg^2+^ homeostasis are unclear (Tomita et al., 2017). Additionally, MgCO_3_ could also provide HCO_3_^−^ to carboxylate PEP and maintain pH in the fermentation broth.

Although the regulation of the SA metabolism process is very complex, many factors could be considered to balance the metabolic process when the strains are modified to achieve efficient production of SA. As is shown in Table 2, the performance of engineered strains constructed by metabolic engineering to produce SA and fix CO_2_ is summarized.

**TABLE 2:** Performance of engineered strains.

Strains	Description/Genetic modification	Substrates	Titer (g/L)	Yield (g/g)	Productivity (g/L/h)	CFR (g/L/h)	References
*Escherichia coli*							
AS1600a	∆ l*dhA*, ∆ *adhE*, ∆ *ackA*, ∆ (*focA-pflB*) ∆ *mgsA*, ∆ *poxB*, ∆ *tdcDE*, ∆ *citF*, ∆ *aspC*, ∆ *sfcA*, pck*, ptsI*	Xylose and glucose	84.3	0.880	0.960	0.358	Sawisit et al. (2015)
SD121	∆ *ldhA*, ∆ *pflB*, ∆ *ptsG*; expression of *ppc*	Xylose mother liquor	52.1	0.630	0.620	0.231	Wang et al. (2014b)
Tang1527	∆ *ldhA*, ∆ *pflB*, ∆ *ptsG*; expression of *bicA*, *sbtA*, *ppc* and *pck*	Glucose	89.4	1.24	1.24	0.463	Yu et al. (2016)
Tang1541	∆ *ldhA*, ∆ *pflB*, ∆ *ptsG*; cra*	Glucose	79.8	0.790	0.990	0.369	Zhu et al. (2016)
YL104H	∆ *poxB*, ∆ *pta*, ∆ *iclR*, ∆ *sdhA*, ∆ *arcA*, ∆ *adhE*, ∆ *ptsH*, ∆ *ldhA*, ∆ *ptsG*	Xylose mother liquor	61.7	—	0.950	0.355	Zhang et al. (2016)
*Mannheimia succiniciproducens*							
PALK	∆ *ldhA*, ∆ *pta*, ∆ *ackA*	Glucose and glycerol	90.7	0.750	3.49	1.30	Choi et al. (2016)
PALFK	∆ *ldhA*, ∆ *pta*, ∆ *ackA*, ∆ *fruA*	Sucrose and glycerol	78.4	1.07	6.02	2.24	Lee et al. (2016)
PALKG	∆ *ldhA*, ∆ *pta*, ∆ *ackA*; expression of *glpK*	Sucrose and glycerol	64.7	0.910	3.34	1.25	Lee et al. (2016)
*Corynebacterium glutamicum*							
K3	∆ *ldh*, ∆ *pta-ackA*, ∆ *cat*; overexpression of *pyc*, *ppc* and *Ncgl0275*	Corn stover	118	0.590	1.04	0.388	Li et al. (2023b)
NC-3-1	∆ *ldhA*; expression of *xylAB*, *gapA*	Glucose	113	0.940	2.34	0.878	Xu et al. (2016)
*C.g*1006	∆ *ldhA*	Cane molasses	35.1	0.0800	0.650	0.243	Xu et al. (2015)
*Yarrowia lipolytica*							
PGC01003	∆ *Ylsdh5*	Glycerol	51.9	0.420	1.46	0.545	Li et al. (2017a)
PSA02004	∆ *Ylsdh5*; adaptive evolution of PGC01003	Food waste	87.9	0.560	0.700	0.261	Yang et al. (2017)
PSA02004PP	overexpression of *xr*, *xdh* and *xk* in PSA02004	Xylose	11.2	0.190	0.120	0.0435	Prabhu et al. (2020)
Y-3314	∆ *ach*, expression of *pck* and *scs2*	Glycerol	111	0.530	0.800	0.299	Cui et al. (2017)
*Synechococcus elongatus* PCC							
CR8/PCC7942	Ptrc: *gabD*, *kgd*, *gltA*, *ppc*; SpecR integrated at NSI and PLlacO1: d*cas9*; P*trc*: sgRNA (*glgC*-1); P*trc*: sgRNA (*sdhB*-2); KanR integrated at NSII in PCC7942 chromosome	Ambient CO_2_	8.90	—	0.170 g/L/d	0.0639 g/L/d	Lai et al. (2022)

Notes: CFRs (CO_2_ fixation rates) were approximately calculated from the related references.

## 3 Production of SA from waste biomass

As a renewable feedstock for the production of SA, the potential that biomass shows for carbon sequestration is also often discussed (Forfora et al., 2024). When compared to fossil-fuel-based chemicals, bio-based chemicals produced by biomass are predicted to reduce CO_2_ emissions (Hepburn et al., 2019). The hydrolysate of biomass also provides the feedstock for microbial capture, fixation, and sequestration of carbon dioxide. It usually requires three stages to produce SA from waste biomass. Firstly, the biomass needs to be pretreated to break its difficult-to-hydrolyze structure, and then the pre-treated biomass is enzymatically hydrolyzed to fermentable sugars, which could finally be fermented to SA (Narisetty et al., 2022; Pakchamni et al., 2022). Pretreated biomass could be used to produce SA by separate hydrolysis and fermentation (SHF) or by one-pot reaction methods such as simultaneous saccharification and fermentation (SSF), simultaneous saccharification and co-fermentation (SSCF), and consolidated bioprocessing (CBP) (Lu et al., 2021; Okolie et al., 2022). This section describes the enzymes required for the production of SA and the hydrolysis of waste biomass after relevant pretreatment.

### 3.1 Waste biomass pretreatment and utilization

Pretreatment of biomass includes physical, chemical, and biological methods. Alkali treatment is simple and effective and is a commonly used chemical treatment. For carbohydrate-rich sugarcane bagasse, alkali treatment with NaOH and enzymatic hydrolysis resulted in fermentable sugar yields of up to 88.7%. SA production using sugarcane bagasse hydrolysate was conducted, in which SA titer and yield were 33.2 g/L and 0.580 g/g, respectively (Ong et al., 2019). Additionally, sugarcane bagasse pre-treatment with alkaline hydrogen peroxide (AHP) at mild conditions could obtain a yield of 74.3% fermentable sugar. Followed by fed-batch fermentation to produce SA, all glucose and xylose could be utilized, and the obtained concentration and yield of SA reached 41.4 g/L and 0.290 g/g sugarcane bagasse raw material (Zhang et al., 2022).

Ionic liquids (ILs) are a promising biomass pretreatment solvent with the advantages of being non-toxic, stable, and recyclable. Under relatively mild conditions, ILs can effectively dissolve cellulose, break the dense structure formed by lignin, and expose cellulose from the coating of lignin and hemicellulose. Pinewood was pretreated with 1-allyl-3-methylimidazolium chloride [AmimCl], and the enzymatic hydrolysis rate of pinewood extract could reach 72.2%. *A. succinogenes* 130Z can produce 20.7 g/L SA from pinewood extract with a CFR of 0.336 g/L/h (Wang C. et al., 2014). Additionally, mulberry stem (MS) was pretreated with cholinium-glycinate ([Ch][Gly]) and cholinium-alanate ([Ch][Ala]). Compared to untreated samples, glucose yield increased from 14.0% to 74.0%. *A. succinogenes* ATCC55618 was applied for SA fermentation with a productivity of 1.18 g/L/h and a CFR of 0.440 g/L/h (Pakdeedachakiat et al., 2019). In addition to lignocellulose, microalgal biomass could also be pretreated by ILs (Sorokina et al., 2020). With 1-butyl-3-methylimidazolium hydrogen sulfate ([BMIM][HSO4]), microalgal biomass was treated directly by biomass transesterification. The sugar yield of microalgae extract after acid hydrolysis was 81.1%. Being fermented with A. succinogenes 130Z, the purified hydrolysate obtained a SA productivity of 0.190 g/L/h and a CFR of 0.0708 g/L/h. In fact, microalgae themselves also have strong carbon sequestration capacity (Chiang et al., 2021), which also increases the effect of carbon capture, utilization and storage (CCUS) in the SA production process (Yang et al., 2022; Xu et al., 2023).

Corn fiber hydrolysate could be obtained by using optimized liquid hot water (LHW). Being fermented by *A. succinogenes* 130Z, corn fiber hydrolysate could produce 27.8 g/L SA with a CFR of 0.288 g/L/h (Vallecilla-Yepez et al., 2021). The sugarcane bagasse residue obtained by LHW and alkali-pretreated has better digestibility and a higher SA conversion rate. And Chen et al. developed an *in-situ* semi-simultaneous saccharification and co-fermentation process to produce SA from sugarcane bagasse, which could obtain 41.0 g/L SA with a CFR of 0.0921 g/L/h (Chen et al., 2021). Hassan et al. pretreated the oil palm empty fruit bunch (OPEFB) with inorganic salts to improve its delignification and saccharification yields and obtained a large amount of total reducing sugar (Hassan et al., 2020). Based on the inorganic salt pretreatment of OPEFB, SA is produced through SSF, with a production of 65.2 g/L and a yield of 0.650 g/g OPEFB (Khairil Anwar et al., 2021). Hydrolysis products obtained by dilute acid and enzyme pretreatment of durian shells were recently reported to be used as a carbon source for succinic acid production, with product yields of 49.0% and 63.0%, respectively. The fermentation of durian shell hydrolysate consumes 0.280–0.310 kg of carbon dioxide per kg of succinic acid produced, which is a potential technology for the production of carbon sequestration and high value-added chemicals (Woo et al., 2023). Therefore, a gentle approach to biomass pretreatment is key to the green manufacture of bio-based chemicals.

The biosynthesis of SA from biomass using mild pretreatment, enzymatic saccharification, and microbial fermentation provides an efficient and sustainable replacement for petroleum-based methods (Zhang et al., 2022). Additionally, oil palm trunk (OPT) juice could also be used as the sole carbon source to produce SA. It was demonstrated that when using OPT sap as the only substrate, the SA yield and productivity could reach 0.540 g/g and 0.350 g/L/h (Bukhari et al., 2019). This work shows that OPT sap contains enough nutrients for *A. succinogenes* 130Z to synthesize SA, and could reduce costs without the supplement of expensive nutrients. There are many cheap raw materials available to produce SA, such as mixed food waste and vegetable waste (Li C. et al., 2018; Li et al., 2019), and how to reduce costs while increasing the yield of products is still a problem to be considered in industrial production.

### 3.2 Hydrolytic enzymes for waste biomass

Waste biomass is a plentiful and cheap renewable carbon source, including agricultural residues such as sugarcane bagasse and wheat straw, as well as food waste generated by human activities (Pati et al., 2023). The main components of most of these biomasses are cellulose, hemicellulose, and lignin (Zerva et al., 2021). Naturally, a wide range of microorganisms carry a rich and sophisticated enzymatic arsenal, including cellulase, hemicellulase, ligninase, and auxiliary enzymes, which could completely degrade waste biomass into fermentable sugars (Guo H. et al., 2022; Guo X. et al., 2022). These hydrolytic enzymes have been extensively studied (Guo et al., 2023), such as cellulase in *Bacillus tequilensis* (ON754229) (Malik and Javed, 2024), hydrolase in cellulolytic nitrogen-fixing bacteria (Harindintwali et al., 2022), and β-glucosidase and xylanase in *Trichoderma asperellum* LYS1 (Mou et al., 2023). Newly identified hydrolases with excellent enzymatic properties could be obtained from nature, but the process is time-consuming and labor-intensive. In recent years, with the continued refinement of protein structure analyses and high-throughput screening, directed evolution, semi-rational and rational design have been used to rapidly improve the thermostability, catalytic activity, and substrate specificity of hydrolases (Dadwal et al., 2020).

Although numerous microorganisms applied to biomass hydrolysis have been investigated, microorganisms which are cellulolytic and SA-producing at the same time have rarely been reported. Yang et al. constructed an engineered yeast strain carrying the gene encoding endo-polygalacturonase from *Aspergillus niger* 1805 to produce oligogalacturonides directly from citrus peel wastes (Yang et al., 2020). Fathima et al. employed *Clostridium phytofermentans DSM1183* to directly bioconvert waste water algal biomass into ethanol (Fathima et al., 2016). According to studies that bioconverting waste biomass into high-value compounds by hydrolase-producing strains, it may be possible to achieve a one-pot biosynthesis from waste biomass to SA by integrating the functions of hydrolase-producing strains and SA-producing strains. For instance, genes encoding hydrolytic enzymes could be introduced into SA-producing engineered strains, or cellulolytic microorganisms could be given the ability to produce SA, which could enable strains to produce SA directly from biomass. Moreover, hydrolase-producing microorganisms and SA-producing microorganisms could be co-cultured and fermented to realize one-pot biosynthesis from waste biomass to SA.

## 4 Process improvement for improved SA yield and carbon sequestration

### 4.1 Bioelectrochemical strategies

NADH is often required to provide reducing power during anaerobic fermentation of SA, for which an external potential supply can provide more energy required for the enzyme-catalyzed reaction, thus improving the SA biosynthesis process. Different levels of oxidation-reduction potential (ORP) were examined in SA production from glucose by *E. coli* to determine the optimal external potential supply (Liu et al., 2014). Since the ORP level affects enzyme activity and metabolic flux, cell growth and SA yield change with the ORP level ranging from −200 to −400 mV during anaerobic fermentation. At the redox potential level of −400 mV, SA production reached 28.6 g/L, with a yield increased by 39% compared to that of −200 mV. Amulya et al. also applied the bioelectrochemical system (BES) to SA production from CO_2_ by facultative anaerobic bacteria *Citrobacter amalonaticus* (Amulya and Venkata Mohan, 2021). When the constant applied potential was −800 mV, SA yield reached 14.4 g/L. In this context, the replenishment of external potential could effectively maintain the microbial redox reactions in the fermentation system and is especially conducive to the regeneration of NAD(H) (Barin et al., 2023).

Additionally, a novel electrochemical membrane bioreactor was designed to produce SA, which enables the *in-situ* separation of SA in the anode chamber through an anion exchange membrane (Stylianou et al., 2023). With the organic fraction of municipal solid waste (OFMSW) as feedstock, this electrochemical and bioreactor coupling strategy could produce SA 66.7 g/L with a yield of 0.510 g/g. In this electrochemical process, compared to the traditional process, the use of renewable power could replace the use of alkali and acid, increase SA productivity, and reduce SA purification operations.

### 4.2 Bioreactions coupling system

The primary benefit of SA production in anaerobic conditions is the ability of CO_2_ fixation during the conversion of PEP or pyruvate to oxaloacetate. When compared to fossil-fuel-based SA, each ton of bio-based SA is predicted to reduce CO_2_ emissions by 4.50–5.00 tons (Gunnarsson et al., 2014). Therefore, the generation of off-gases can be coupled with a fermentation-synthesis process to capture and utilize CO_2_ more efficiently.

Linked-fermentation strategies can help to produce multiple products at once and the substrates can be more fully utilized (Zhang et al., 2017; Gao et al., 2022; Yin and Wang, 2022). *Saccharomyces cerevisiae* and *Actinobacillus succinogenes* were co-cultured in the hydrolysate of sugarcane bagasse. *S. cerevisiae* produced EtOH and CO_2_ from glucose in the hydrolysate, and *A. succinogenes* used reducing sugars in the hydrolysate and CO_2_ to synthesize SA. After fermentation in a 1.5-L fermenter for 60 h, 22.0 g/L of EtOH and 22.1 g/L of SA were obtained, with a CFR of 0.137 g/L/h (Xu et al., 2021). Hence, combining the production of ethanol (EtOH) and SA could not only reduce CO_2_ emissions, but also supply a significant amount of CO_2_ for SA production.

There are other similar substrate co-fermentation strategies, such as co-production of SA and cadaverine (Gao et al., 2022). The SA pathway was introduced into cadaverine-producing strains, and a thermal switch system was established and optimized to realize a two-stage co-production. Cell proliferation and lysine synthesis occurred during the primary stage, while SA and cadaverine were produced during the following stage. In a 5 L bioreactor, the production of SA reached 28.4 g/L, while that of cadaverine was 55.6 g/L. In the whole process, decarboxylation of L-lysine to form cadaverine releases CO_2_, while carboxylation of PEP to form oxaloacetate fixes CO_2_ (Xi et al., 2022). Based on this fact we conclude that CO_2_ emissions could be greatly decreased during co-production, which would benefit the atom economy.

Additionally, a two-chamber bioreactor coupling device was constructed to produce EtOH and SA respectively and the CO_2_ generated from EtOH fermentation was directly supplied to SA fermentation. For instance, the CO_2_ generated in EtOH fermentation from corn was absorbed on a packed column that contained a solution of NaOH, KOH, or NH_4_OH. The obtained carbonate solution was used both for pH control and for the supply of CO_2_ for SA production (Nghiem and Senske, 2015). By optimizing the volume ratio of the working medium in the two-chamber bioreactor, the maximum yields of EtOH and SA from glucose were 0.510 g/g and 0.700 g/g, respectively. As a result, after fermentation in a 3 L fermenter for 70 h, 31.6 g/L of SA was obtained with a CFR of 0.168 g/L/h (Qian and Zheng, 2023).

Amulya et al. screened a *Citrobacter amalonaticus* to produce SA and studied its fixation of CO_2_ (Amulya and Mohan, 2019). They used H_2_ gas as an electron donor, and simultaneously externally supplied H_2_ and CO_2_ gases for fermentation during SA production. With sucrose as substrate, *Citrobacter amalonificus* could produce SA 12.1 g/L with a CO_2_ partial pressure of 1 bar. Furthermore, increasing CO_2_ partial pressure from 0.6 to 2 bar resulted in an increase from 0.210 to 0.460 g/L/h of SA productivity, and CFR increased from 0.0783 to 0.172 g/L/h (Amulya et al., 2020). Moreover, the production of the by-products under high-pressure CO_2_ conditions was significantly reduced, especially lactic acid and formic acid (Mota et al., 2019). Carbonate replaced with CO_2_ at different ratios also improves the yield and selectivity of SA, as gaseous CO_2_ penetrates cell membranes and is better utilized by cells (Yang et al., 2024). Therefore, it is believed that the most important factors affecting SA generation are the solubility of CO_2_ and extra electron donors (Tan et al., 2014). The positive impact of CO_2_ on the production of SA creates opportunities for sustainable collaboration between SA industries and other biofuel industries that generate CO_2_ (Annie Modestra et al., 2020). Although the additional supply of CO_2_ can increase SA production, commercial CO_2_ is relatively expensive and not suitable for industrial production. From the perspective of industrial application, the linked-fermentation strategy is more environmentally friendly and has more application potential than traditional SA fermentation (Su et al., 2021). However, the CO_2_ provided by the linked-fermentation strategy is limited, and the supply may be unstable, so the industrial production of SA with CO_2_ as the sole fermentation substrate remains a great challenge.

## 5 Techno-economic analysis and carbon sequestration benefits

The cost of feedstock significantly influences the economics of the manufacturing of bio-based SA (Stylianou et al., 2020). The use of CO_2_ and waste biomass as feedstocks to produce SA has high economic benefits and the production cost is also affected by the options of alkaline neutralizers. As mentioned above, although MgCO_3_ has better performance in fermentation, its high cost is not suitable for SA biorefineries. Replacing MgCO_3_ with a mixture of low-cost Mg(OH)_2_ and NH_3_·H_2_O could also obtain high SA yields (Wang J. et al., 2014).

A multiproduct model using pulp logs as biomass feedstock was studied, producing acetate, dimethyl ether and SA (Ghayur et al., 2019). In terms of the capacity to mitigate climate change, the advantage is that acetate is biosynthesized from biomass rather than being derived from fossil fuel, and dimethyl ether could be used for power generation. In terms of economy, the additional revenue from the sale of acetate and dimethyl ether compensates for the production costs. Therefore, a multiproduct SA biorefinery is technically and economically feasible, with the potential to be carbon-negative and to stimulate regional economics. The novel SA biorefinery could make full use of all the reducing sugars from the waste biomass to produce a wide range of chemicals, including SA, with CO_2_ as one of the feedstocks to produce the platform chemicals (Kumar et al., 2022). Therefore, the production of chemicals in biorefineries is also a process of CO_2_ integration, which has technical, financial and environmental advantages (Filippi et al., 2022).

Another simulation process is to produce SA from glycerol and add dimethyl sulfoxide as the electron acceptor (Thanahiranya et al., 2023). The model evaluates the performance of the selected situations according to techno-economic, efficiency, and greenhouse gas emissions. The results demonstrate that adding dimethyl sulfoxide to fermentation is the key to produce SA, which solves the problem of excessive glycerol (Carvalho et al., 2014; Kaur et al., 2020), and the best profit expected to be generated over 5 years is $190 million, with an internal rate of return of 33.3%. The next compelling step is to set up a pilot manufacturing plant to validate and optimize the models to reduce the risk of new synergies required for such approaches.

Several types of research have been done to investigate the environmental benefits of SA synthesis from waste biomass such as bread waste, the organic fraction of municipal solid waste (OFMSW), apple pomace, etc. The environmental impact of SA produced from waste bread is substantially lower than that of SA derived from fossil fuels. Furthermore, although the emissions of greenhouse gas were relatively higher compared to corn and sorghum grains, using waste bread eliminates the requirement for food crops and arable land (Gadkari et al., 2021). Additionally, OFMSW could also be used as a feedstock for SA fermentation as it contains 30.0%–60.0% carbohydrates (Stylianou et al., 2021). Considering the CO_2_ emissions from OFMSW landfill disposal, SA biorefining from OFMSW contributes 35.0% fewer CO_2_ emissions than traditional processes based on fossil-derived. Moreover, the established biorefinery generated sugar-rich hydrolysate with 100% recovery of oils/fats and 68.0% recovery of protein, which was then used to produce SA (Ladakis et al., 2022). This cradle-to-gate life cycle assessment (LCA) approach is also typically used for techno-economic analysis of chemical production to determine its environmental impact (Ögmundarson et al., 2020). As is shown in Table 3, the economic and carbon sequestration benefits of various SA biorefineries are listed.

**TABLE 3:** Economic and carbon sequestration benefits for SA.

Feedstocks	SA minimum selling price ($/t)	Best profit ($/y)	CO_2_ sequestration (t/y)	References
Pulp logs	9.90 × 10^2^	incalculable	4.20 × 10^4^	Ghayur et al. (2019)
Glycerol	4.50 × 10^2^	4.24 × 10^7^	3.73 × 10^3^	Thanahiranya et al. (2023)
OFMSW	2.94 × 10^3^	1.41 × 10^8^	1.87 × 10^4^	Stylianou et al. (2021)
OFMSW	1.13 × 10^3^	incalculable	2.24 × 10^4^	Ladakis et al. (2022)
Winery waste	1.23 × 10^3^	2.92 × 10^7^	1.13 × 10^4^	Ioannidou et al. (2022)
Corn stover	1.70 × 10^3^	2.86 × 10^6^	1.12 × 10^4^	Dickson et al. (2021)
Sugarcane bagasse	2.37 × 10^3^	3.24 × 10^7^	1.49 × 10^4^	Shaji et al. (2021)

Notes: Minimum Selling Price (MSP) is the minimum sale price acceptable to the authority based on current market value and sales price data. Best profit is the maximum profit that can be made each year. CO_2_ sequestration was calculated approximately based on the information offered in the relevant references.

The techno-economic analysis, life cycle assessment and carbon sequestration benefit evaluation of SA refineries are conducive to advancing the industrialization of SA biosynthesis. However, the energy consumed by SA biorefineries and the subsequent separation and purification processes are still the main obstacles to the large-scale production of bio-based SA. Although the fixation of CO_2_ for SA production can achieve high carbon sequestration benefits, a stable industrial supply of CO_2_ is still an unsolved problem. Owing to the above-mentioned disadvantages of bio-based SA, two suggestions could be proposed. One is to construct a more robust microbial host for SA fermentation with high yields and productivity at low CO_2_ supply. Another is to develop a fermentation process strategy with low-cost raw materials as the primary substrate and CO_2_ as a secondary substrate, which can help to realize a process with high economic competitiveness (Li et al., 2021). Bio-based products biorefineries would be more desirable and might eventually take the place of petroleum-based production if a method with higher energy efficiency was developed.

## 6 A new technology to assist SA biosynthesis—droplet microfluidics-based high-throughput screening

Currently, there are still some problems to be solved for SA biosynthesis, such as the fact that most succinic acid-producing strains prefer neutral pH conditions, but succinic acid formation acidifies the medium, leading to low robustness of the engineered bacteria and high cost of recovery of downstream products; and low activity of enzymes for hydrolyzing biomass. All these problems also limit the CO_2_ fixation during SA synthesis. To address the above problems, screening engineered strains with high SA production and acid tolerance by high-throughput screening techniques is a promising approach, taking efficiency and cost into account.

Conventional high-throughput screening platforms use 96 microplates, based on the principles of “arrangement” and “combination,” to systematically perform basic fluidic operations through automation, followed by detection and data processing to complete the analysis process. One or more high-yielding strains can be screened from a library of mutant strains (Hertzberg and Pope, 2000). Although the screening flux is indeed greatly improved compared to manual screening of strains, there are also major constraints, such as expensive instruments, inability to avoid cross-contamination, and a large difference in the efficiency and cost of data collection (Shi et al., 2023). To address the above problems, it is revolutionary to use droplet microfluidics instead of well plates to realize high-throughput screening. Compared to high-throughput screening with well plates, droplet microfluidics not only improves the flux by at least 4 orders of magnitude, but also drastically reduces the cost and time of each experiment (Ding et al., 2020).

Therefore, if the high-throughput screening platform of droplet microfluidics can be used for the screening of key enzymes or strains in SA biosynthesis, it will undoubtedly accelerate the development of SA biosynthesis. Based on this, the combination of ultra-high-throughput droplet microfluidic screening platforms and spectroscopic techniques also provides for high-throughput screening of strains, such as Raman-activated cell sorting (RACS) (Nitta et al., 2020) and fluorescence-activated cell sorting (FACS) (Li C. et al., 2023). Combining a droplet-captured microfluidic cell-sorting device with a spectral analysis device allows individual cells to be analyzed by Raman microspectroscopy or fluorescence detection, and cells can be sorted as needed. By using in-depth computational analysis strategies and artificial intelligence, new enzymes or pathways for SA biosynthesis can be discovered and constructed, with AI-designed enzyme mutants often exhibiting higher activity and stability (Li R. et al., 2018; Gargiulo and Soumillion, 2021). The development of machine learning algorithms, such as AlphaFold2, has also facilitated the analysis of enzyme structures and properties, allowing the prediction of beneficial mutation sites based on the structure of key enzymes (Fang et al., 2022; Ge et al., 2023). In addition, the construction of SA-responsive biosensing cells may be the key to realizing high-throughput screening of SA-producing strains, and thus the development of SA-responsive bioelements is imminent. Based on this, we are recently designing a droplet microfluidic screening platform based on surface-enhanced Raman spectroscopy in order to realize high-throughput screening of strains with high SA production. This system, if successfully realized, will achieve detection and sorting of about 2 million single drops at 160 drops/s, which will be a great innovation. In addition, high-yielding SA-producing strains exhibit greater CO_2_ fixation capacity, leading to higher carbon sequestration benefits.

Taken together, these strategies help to select the best enzyme or strain mutant in the shortest possible time, creating a great opportunity for the modification of engineered strains to produce SA for utilization of CO_2_ and waste biomass as feedstock, and achieving higher carbon sequestration benefits.

## 7 Conclusion

The biosynthesis process of bio-based SA has been attracting attention. The rise of SA biorefineries from CO_2_ and waste biomass as feedstocks shows great potential for CO_2_ emission reduction and environmental protection. By exploring novel technologies of synthetic biology and integrating multiple disciplines, such as biosensing and artificial intelligence, the biosynthesis strategy could establish a green and sustainable future for SA production, achieve great techno-economic feasibility and CO_2_ sequestration efficiency, and result in harmony between man and the natural environment.
